# Monocyte chemoattractant protein-1 serum levels in ovarian cancer patients

**DOI:** 10.1038/sj.bjc.6690776

**Published:** 1999-11

**Authors:** L Hefler, C Tempfer, G Heinze, K Mayerhofer, G Breitenecker, S Leodolter, A Reinthaller, C Kainz

**Affiliations:** Departments of Gynaecology and Obstetrics, Medical Computer Sciences andGynaecopathology, University of Vienna Medical School, Währinger Gürtel 18–20, Vienna, A-1090, Austria; Department of Obstetrics & Gynaecology, Houston, Texas, USA

**Keywords:** MCP-1, ovarian cancer, prognosis, differentiation marker

## Abstract

The chemokine monocyte chemoattractant protein (MCP)-1 is an important mediator of monocyte infiltration in various solid tumours of epithelial origin. The aim of the present study was to evaluate the role of MCP-1 in the natural history of ovarian cancer and to determine its value as differentiation marker and prognostic marker regarding disease free and overall survival. This retrospective study comprises 86 patients with ovarian cancer, 48 with primary ovarian cancer and 38 with recurrent ovarian cancer, 67 patients with benign ovarian cysts and 42 healthy women. Median serum levels in patients with primary ovarian cancer, recurrent ovarian cancer, benign ovarian cysts and in healthy women were 535.6 (range 129.6–1200) pg ml^–1^, 427.3 (range 193.4–1101) pg ml^–1^, 371.2 (range 222–986.8) pg ml^–1^ and 318.7 (range 241.3–681.4) pg ml^–1^ respectively (Mann–Whitney *U*-test, *P* < 0.001). Univariate logistic regression models revealed a significant influence of MCP-1 serum levels on the odds of presenting with primary ovarian cancer versus benign cysts and versus healthy women respectively (univariate logistic regression, *P* < 0.001 and *P* < 0.001 respectively). In a multivariate logistic regression model considering MCP-1 and CA 125 serum levels simultaneously, both MCP-1 and CA 125 revealed statistical significance on the odds of presenting with primary ovarian cancer versus benign cysts (multivariate logistic regression, *P* = 0.05 and *P* < 0.001 respectively). In ovarian cancer patients, MCP-1 serum levels showed a statistically significant correlation with histological grade (Mann–Whitney *U*-test, *P* = 0.02) and age at the time of diagnosis (Mann–Whitney *U*-test, *P* = 0.03). Elevated MCP-1 serum levels prior to therapy were not associated with disease-free and overall survival (log-rank test, *P* = 0.2 and *P* = 0.7 respectively). In summary these data indicate that MCP-1 might play a functional role in the natural history of ovarian cancer and might serve as differentiation marker between benign ovarian cysts and ovarian cancer, providing additional information to the established tumour marker CA 125. © 1999 Cancer Research Campaign


British Journal of Cancer (1999) 81(5), 855–859
© 1999 Cancer Research Campaign
Article no. bjoc.1999.0776

### Monocyte chemoattractant protein-1 serum levels in ovarian cancer patients

**L Hefler[1], C Tempfer[4], G Heinze[2], K Mayerhofer[1], G Breitenecker[3], S Leodolter[1], A Reinthaller[1] and C Kainz[1]**

Departments of [1]Gynaecology and Obstetrics, [2]Medical Computer Sciences and [3]Gynaecopathology, University of Vienna Medical School, Währinger Gürtel 18–20, A-1090 Vienna, Austria;; [4]Department of Obstetrics & Gynaecology[2] Baylor College of Medicine, Houston, Texas, USA

**Summary** The chemokine monocyte chemoattractant protein (MCP)-1 is an important mediator of monocyte infiltration in various solid tumours of epithelial origin. The aim of the present study was to evaluate the role of MCP-1 in the natural history of ovarian cancer and to determine its value as differentiation marker and prognostic marker regarding disease free and overall survival. This retrospective study comprises 86 patients with ovarian cancer, 48 with primary ovarian cancer and 38 with recurrent ovarian cancer, 67 patients with benign ovarian cysts and 42 healthy women. Median serum levels in patients with primary ovarian cancer, recurrent ovarian cancer, benign ovarian cysts and in healthy women were 535.6 (range 129.6–1200) pg $ml^{-1}$, 427.3 (range 193.4–1101) pg $ml^{-1}$, 371.2 (range 222–986.8) pg $ml^{-1}$ and 318.7 (range 241.3–681.4) pg $ml^{-1}$ respectively (Mann–Whitney *U*-test, $P < 0.001$). Univariate logistic regression models revealed a significant influence of MCP-1 serum levels on the odds of presenting with primary ovarian cancer versus benign cysts and versus healthy women respectively (univariate logistic regression, $P < 0.001$ and $P < 0.001$ respectively). In a multivariate logistic regression model considering MCP-1 and CA 125 serum levels simultaneously, both MCP-1 and CA 125 revealed statistical significance on the odds of presenting with primary ovarian cancer versus benign cysts (multivariate logistic regression, $P = 0.05$ and $P < 0.001$ respectively). In ovarian cancer patients, MCP-1 serum levels showed a statistically significant correlation with histological grade (Mann–Whitney *U*-test, $P = 0.02$) and age at the time of diagnosis (Mann–Whitney *U*-test, $P = 0.03$). Elevated MCP-1 serum levels prior to therapy were not associated with disease-free and overall survival (log-rank test, $P = 0.2$ and $P = 0.7$ respectively). In summary these data indicate that MCP-1 might play a functional role in the natural history of ovarian cancer and might serve as differentiation marker between benign ovarian cysts and ovarian cancer, providing additional information to the established tumour marker CA 125.

**Keywords:** MCP-1; ovarian cancer; prognosis; differentiation marker

Monocyte chemoattractant protein (MCP)-1, a 76-amino acid protein, is a member of the C-C subfamily of chemokines. MCP-1 has been shown to cause monocyte and macrophage recruitment during inflammation states (Grimm et al, 1996; Mazzucchelli et al, 1996). Furthermore, MCP-1 has been implicated in mediating monocyte and tumour-associated macrophage (TAM) infiltration in various solid tumours of epithelial origin (Leonard et al, 1990). The role of TAM infiltration of malignant tumours is still controversial. TAMs have been found to be cytotoxic to tumour cells when activated in vivo or in vitro (Fidler et al, 1984; Brunda et al, 1991). However, TAMs constitute a heterogeneous population that may have both inhibitory and stimulatory influences on malignant growth (Peng et al, 1997). Mantovani et al (1994) reported that TAMs promote tumorigenesis, angiogenesis and metastatic spread of malignant tumours by producing various chemokines such as transforming growth factor (TGF)-β, TNF-α, interleukin (IL)-1 and IL-6.

MCP-1 has been investigated in a wide variety of human malignancies, e.g. malignant glioma, melanoma, bladder, prostate and breast cancer (Graves et al, 1992; Kuratsu et al, 1993; Desbaillets et al, 1994; Mazzucchelli et al, 1996; Amann et al, 1998; Valkovic et al, 1998). Regarding ovarian cancer, Negus et al (1995) reported that MCP-1, as produced by epithelial ovarian cancer cells, contributes to the local accumulation of TAMs, consequently influencing tumour behaviour. Furthermore, MCP-1 down-regulation correlates with inhibition of monocyte migration within ovarian cancer tissue (Negus et al, 1998). To the authors' knowledge no data on MCP-1 serum levels in ovarian cancer patients have been reported to date.

The aim of this study was to evaluate whether MCP-1 serum levels could serve as differentiation marker for ovarian cancer and benign ovarian cysts, providing additional information to the established tumour marker CA 125. A further aim of the study was to investigate the prognostic potential of MCP-1 serum levels regarding disease-free and overall survival. In this respect we examined MCP-1 serum levels in healthy women and in patients with benign cysts, primary ovarian cancer and recurrent ovarian cancer.

### MATERIALS AND METHODS

### Patient characteristics

This retrospective study includes 86 serological examinations of patients with ovarian cancer. Clinical data were obtained from files at the University Hospital of Vienna, Department of Gynaecology and Obstetrics. Forty-eight patients had primary ovarian cancer International Federation of Gynaecology and Obstetrics (FIGO) stages I ($n = 8$), II ($n = 11$) and III ($n = 29$). Median age at the time

*Received 16 September 1998*
*Revised 23 March 1999*
*Accepted 30 March 1999*

*Correspondence to:* L Hefler of diagnosis was 59 (range 29–87) years. Histologically, 21 tumours were typed as serous adenocarcinoma, 14 as mucinous adenocarcinoma, 7 as undifferentiated carcinoma, 3 as clear cell carcinoma and three as endometrioid ovarian cancer. Thirty-eight patients had recurrent ovarian cancer.

Additionally, we investigated MCP-1 serum levels in 67 patients with benign cysts. Serous and mucinous cystadenomas were found in 45 and 22 cases respectively. Furthermore, MCP-1 serum levels were evaluated in a panel of 42 healthy women. CA 125 levels were evaluated in all patients with primary ovarian cancer and benign cysts.

### Clinical management

Patients with primary ovarian cancer underwent hysterectomy, bilateral salpingo-oophorectomy, pelvic and para-aortic lymphadenectomy, and omentectomy. Patients with tumour stages Ic–III and patients with clear cell carcinoma received a platinum and/or paclitaxel containing chemotherapy regimen. Patients with recurrent ovarian cancer received cytoreductive surgery and/or a salvage chemotherapy regimen. Histological grading was carried out according to International Union Against Cancer criteria. The stage of disease was classified according to FIGO. All patients were followed up in 3-month intervals including vagino-rectal palpation, abdominal ultrasound examination and serum tumour marker evaluation. In cases of clinically doubtful findings and/or tumour marker elevation computerized tomography was performed. For the aim of this study, all histological specimens were reviewed by an experienced pathologist, blinded to the clinical data.

### Serum assay

Patients' blood was obtained 24–48 h before surgery or at the time of diagnosis of recurrent ovarian cancer by peripheral venous puncture and was immediately centrifuged at 3000 ***g*** for 15 min. The serum was frozen at –80°C until examination. For the measurement of serum MCP-1 a commercially available enzyme-linked immunosorbent assay was used (Quantikine™ Human MCP-1 Immunoassay; R&D Systems, Minneapolis, MN, USA). All serum MCP-1 analyses were performed at the same time, in the same batch, and in duplicate according to manufacturer's instructions. The intra- and inter-assay variability was 4.9% and 5.8% respectively.

### Statistical analysis

Due to the skewed distribution of MCP-1 serum values, median and range of MCP-1 serum levels are given. Comparisons between unpaired groups were made using the Mann–Whitney *U*-test. Survival probabilities were calculated by the product limit method of Kaplan and Meier. Differences between groups were tested using the log-rank test. Spearman correlation coefficients of CA 125 and MCP-1 values were calculated for primary ovarian cancer and benign cysts separately. CA 125 and MCP-1 values were $\log_2$-transformed for further analysis. Logistic regression models were used to analyse the influence of MCP-1 and CA 125 serum levels on the probability for the presence of primary ovarian cancer versus benign cysts. The univariate models show the influence of each of the two variables on the probability for primary cancer without considering the other one. Sensitivity and specificity were calculated for each possible threshold value of estimated probability for primary ovarian cancer versus benign cysts. Based on these values Receiver Operator Characteristics (ROC) curves were constructed to visualize the relationship between MCP-1 and CA 125, respectively, and primary ovarian cancer. Considering CA 125 and MCP-1 simultaneously, multivariate logistic regression analysis was used to compare patients with primary ovarian cancer and benign ovarian cysts. Based on this analysis, a third ROC curve was constructed to show the diagnostic power of their simultaneous consideration. A further univariate logistic regression model was used to analyse the influence of MCP-1 serum levels on the probability for the presence of primary ovarian cancer versus healthy women. Ninety-five per cent confidence intervals (CI) for odds ratios (ORs) were calculated using the profile likelihood method. Odds ratios refer to a unit increase on the $\log_2$-scale, i.e. a doubling on the original scale. *P*-values of ≤ 0.05 were considered statistically significant. We used the SAS statistical software system (SAS Institute Inc., Cary, NC, USA) to do the calculations.

Medical databases MEDLINE since 1966 and EMBASE since 1989 were searched to identify reports on MCP-1 serum concentrations in ovarian cancer patients.

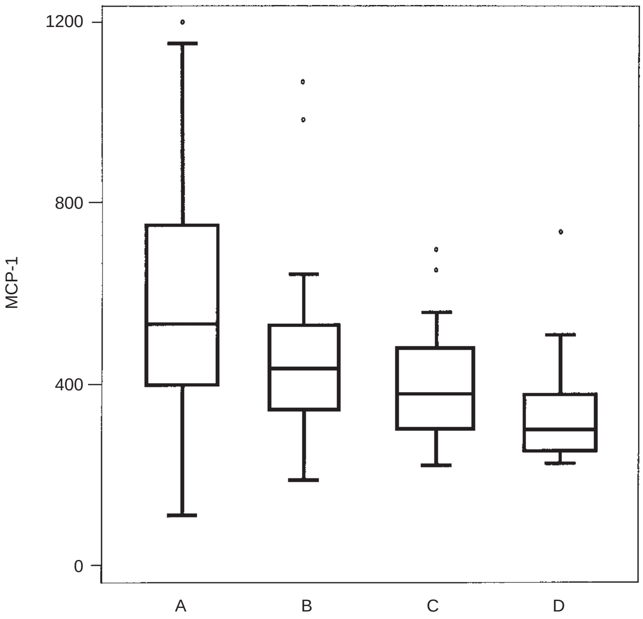

**Figure 1** Box plot of MCP-1 serum levels (pg ml$^{-1}$) in patients with primary ovarian cancer ($n$ = 48; **A**), recurrent ovarian cancer ($n$ = 38; **B**), benign ovarian cysts ($n$ = 67; **C**), and healthy women ($n$ = 45; **D**). Horizontal lines in the box represent the 1st, 2nd (the median), and 3rd quartile; whiskers (vertical lines) represent extension of data up and down, ● represents outside values

### RESULTS

### MCP-1 serum levels in patients with primary ovarian cancer, recurrent ovarian cancer, benign ovarian cysts and healthy women

The overall median MCP-1 serum level was 398.3 (range 129.6–1200) pg ml$^{-1}$. Median MCP-1 serum levels in patients with primary ovarian cancer, recurrent ovarian cancer, benign

 © 1999 Cancer Research Campaign ovarian cysts and healthy women were 535.6 (range 129.6–1200) pg $ml^{-1}$, 427.3 (range 193.4–1101) pg $ml^{-1}$, 371.2 (range 222–986.8) pg $ml^{-1}$ and 318.7 (range 241.3–681.4) pg $ml^{-1}$ respectively. A box plot of MCP-1 serum levels is shown in Figure 1. MCP-1 serum levels did not correlate with CA 125 serum levels in patients with primary ovarian cancer patients (Spearman correlation coefficient, $P = 0.12$) and in patients with benign ovarian cysts (Spearman correlation coefficient, $P = 0.09$).

### MCP-1 serum levels and the presence of primary ovarian cancer and recurrent ovarian cancer compared with healthy women

MCP-1 serum levels were significantly higher in patients with primary ovarian cancer compared with patients with recurrent ovarian cancer (Mann–Whitney *U*-test, $P = 0.02$). Patients with primary and recurrent ovarian cancer showed significantly higher MCP-1 serum levels than healthy women (Mann–Whitney *U*-test, $P < 0.001$ and $P < 0.001$ respectively). In a univariate logistic regression model, MCP-1 serum levels had a significant influence on the odds of presenting with primary ovarian cancer versus healthy women (univariate logistic regression, $P < 0.001$) with an OR of 7.9 (95% CI 3.1–24.1).

### MCP-1 serum levels and the presence of primary ovarian cancer compared with benign cysts

Median MCP-1 serum levels in patients with primary ovarian cancer and benign ovarian cysts were 535.6 (range 129.6–1200) pg $mL^{-1}$ and 371.2 (range 222–986.8) pg $ml^{-1}$ respectively (Mann–Whitney *U*-test, $P < 0.001$). Median CA 125 serum levels in patients with primary ovarian cancer and benign ovarian cysts were 851 (range 13.7–19 619) U $ml^{-1}$ and 15.7 (range 3.1–1340) U $ml^{-1}$, respectively (Mann–Whitney *U*-test, $P < 0.001$). Univariate logistic regression models revealed that MCP-1 and CA 125 serum levels had a significant influence on the odds of presenting with primary ovarian cancer versus benign cysts (univariate logistic regression, $P < 0.001$ and $P < 0.001$ respectively) with ORs of 4.9 (95% CI, 2.3–11.9) and 2.5 (95% CI 1.9–3.6) respectively. At 640 pg $ml^{-1}$ MCP-1 achieved a sensitivity of 31.3% and a specificity of 95%. At 194 U $ml^{-1}$, CA 125 achieved a sensitivity of 65% and a specificity of 95%.

In a multivariate regression model considering serum MCP-1 and CA 125 simultaneously, both MCP-1 and CA 125 revealed a significant influence on the odds of presenting with primary ovarian cancer versus benign cysts (multivariate logistic regression, $P < 0.05$ and $P < 0.001$ respectively). MCP-1 and CA 125 achieved a sensitivity of 75% and a specificity of 95%. ROC curves of MCP-1, CA 125 and the combination of both markers are shown in Figure 2.

### Correlation of MCP-1 serum levels in primary ovarian cancer with tumour stage, lymph node involvement, histological grade and age at the time of diagnosis

When MCP-1 serum levels, taken prior to therapy, were grouped by tumour stage, lymph node involvement, histological grade and age at the time of diagnosis we found a statistically significant correlation with histological grade (357.4 pg $ml^{-1}$ vs 584 pg $ml^{-1}$ for G1 vs G2, G3; Mann–Whitney *U*-test, $P = 0.02$) and age at the time of diagnosis (430.4 pg $ml^{-1}$ vs 582.8 pg $ml^{-1}$ for age < 50 years vs age > 50 years; Mann–Whitney *U*-test, $P = 0.03$). The other investigated parameters showed no significant correlation with MCP-1 serum levels.

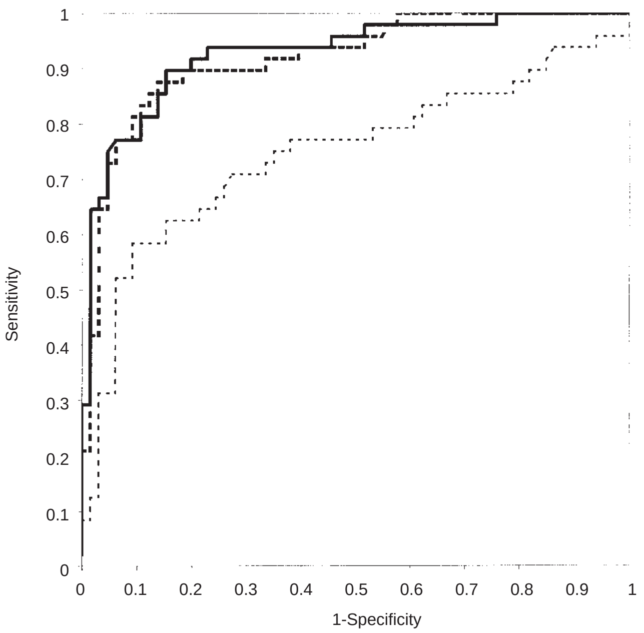

**Figure 2** Receiver operator characteristics (ROC) curves comparing patients with primary ovarian cancer ($n = 48$) and benign ovarian cysts ($n = 67$) with respect to their MCP-1 serum levels (▪▪▪), CA 125 serum levels (■■■) and to a simultaneous consideration of both variables (—)

### Preoperative MCP-1 serum levels as prognostic factors in ovarian cancer

MCP-1 lacks a clearly defined cut-off value. Due to the skewed distribution of MCP-1 serum levels a cut-off value of 718 pg $ml^{-1}$ was selected according to the 75th quantile of serum concentrations measured in the panel of primary ovarian cancer patients. Elevated preoperative MCP-1 serum levels were not associated with disease-free and overall survival (log-rank-test $P = 0.2$ and log-rank-test, $P = 0.7$ respectively).

### DISCUSSION

Chemokines form a superfamily of small, inducible, pro-inflammatory chemotactic cytokines. These proteins have been reported to be involved in a variety of immune and inflammatory responses, acting primarily as chemoattractants and activators of specific leucocytes. MCP-1 is a member of the C-C subfamily of chemokines and is expressed upon stimulation in a wide variety of cells, such as mononuclear leucocytes, fibroblasts, endothelial and epithelial cells, smooth muscle cells, melanocytes and various tumour cells (Schall, 1991). MCP-1 is a key element of the immunological response to malignant growth, mainly via attraction and activation of TAMs (Leonard and Yoshimura, 1990).

Our data show that patients with primary and recurrent ovarian cancer have significantly higher MCP-1 serum levels compared with patients with benign ovarian cysts and healthy women. In our series, MCP-1 had a significant influence on the odds of presenting with ovarian malignancy. This indicates that MCP-1 might be

*© 1999 Cancer Research Campaign*

selectively up-regulated after malignant transformation. MCP-1 seems to play a functional role in the natural history of ovarian cancer, possibly by mediating TAM infiltration of malignant tissue, however, the potential mechanisms involved remain to be elucitated.

Regarding the differentiation between benign and malignant growth, MCP-1 has been investigated in malignant and benign gliomas by Kuratsu et al (1993). They reported that elevated MCP-1 levels in cerebrospinal fluids were indicative of the presence of malignant versus benign glioma. Furthermore, in patients with malignant gliomas, MCP-1 serum levels were associated with subarachnoid dissemination of disease. With respect to these results it has been speculated that MCP-1 might be clinically useful establishing a diagnosis of malignant glioma and in detecting subarachnoidal tumour spread (Kuratsu et al, 1993). With respect to differential diagnosis of benign and malignant adnexal masses, our data showed that MCP-1 serum levels were elevated in patients with ovarian cancer compared with patients with benign ovarian cysts. The higher the MCP-1 serum levels, the higher were the odds of presenting with primary ovarian cancer in our series. A multivariate logistic regression model showed that MCP-1 revealed diagnostic information independent from the established tumour marker CA 125. This documents the fact that MCP-1 is a possible candidate in ongoing efforts to find adequate combined screening systems, e.g. by combining CA 125 with transvaginal ultrasound examination or Doppler ultrasound examination.

The correlation of MCP-1 and histopathological parameters has been investigated in various malignancies. Valkovic et al (1998) reported an association between MCP-1 expression and histological grade in invasive ductal breast cancer. Furthermore, Amann et al (1998) showed that urine MCP-1 levels correlate with histological grade, and distant metastasis in bladder cancer. In accordance with these studies we found lower MCP-1 serum levels in patients with G1 tumours compared with G2 and G3 tumours, whereas MCP-1 serum levels were not associated with tumour stage and lymph node involvement. These findings indicate that MCP-1 serum levels in ovarian cancer are not reflective of the tumour bulk, but rather indicative of strongly proliferating tumours. Consequently, it could be speculated that dedifferentiation of tumour cells provokes a more aggressive host immune response (Negus et al, 1995).

In our study MCP-1 serum levels of patients with primary ovarian cancer and recurrent ovarian cancer were markedly elevated compared with patients harbouring benign ovarian cysts and healthy women. These results are in accordance with experimental data pointing to the involvement of MCP-1 in immunological host defence reactions in ovarian cancer patients (Melani et al, 1995). Additionally, we found a statistically significant difference between primary and recurrent ovarian cancer. Hence, it can be hypothesized that human MCP-1 secretion and host immune response versus malignant growth decreases in the clinical course of ovarian cancer. A decline in immunological competence might be one reason for the worse prognosis of patients with recurrent ovarian cancer compared with patients with primary ovarian cancer (Berek, 1994).

No data have been published so far regarding the prognostic value of MCP-1 serum levels in ovarian cancer. Chen et al (1984) demonstrated that leucocytic infiltration of ovarian cancer tissue is correlated with a favourable prognosis regarding overall survival. Furthermore, Haskill et al (1982) reported that in ovarian cancer patients tumour infiltration by lymphocytes and macrophages is positively associated with response to adjuvant chemotherapy. However, it has also been reported that monocyte/macrophage infiltration of malignant tissue may promote tumour growth by producing various chemokines, e.g. TGF-β, TNF-α, IL-1 and IL-6 (Mantovani et al, 1994; Negus et al, 1995). In our series, MCP-1 serum levels showed no influence on the patients' prognosis regarding disease-free and overall survival.

In summary, our data indicate that MCP-1 might play a functional role in the natural history of ovarian cancer, possibly by mediating host defence reactions, such as TAM infiltration of tumour tissue. Furthermore, MCP-1 is a discriminator between benign adnexal masses and ovarian cancer, providing additional information to CA 125. Further studies are warranted to determine the possible clinical value of MCP-1 in the differential diagnosis of adnexal masses.

### ACKNOWLEDGEMENTS

This study was supported by the Ludwig Boltzmann Foundation, Institute for Gynaecologic Oncology. We thank Mrs Ingrid Schiebel for expert technical assistance.

### REFERENCES

Amann B, Perabo FG, Wirger A, Hugenschmidt H and Chultze-Seeman W (1998) Urinary levels of monocyte chemoattractant protein-1 correlate with tumor stage and grade in patients with bladder cancer. *Br J Urol* **82**: 118–121

Berek J (1994) Epithelial ovarian cancer. In: *Practical Gynecological Oncology*, 2nd edn, Berek J and Hacker N (eds). Williams & Wilkins: Baltimore

Brunda MJ, Sulich V, Wright RB and Palleroni AV (1991) Tumoricidal activity and cytokine secretion by tumor-infiltrating macrophages. *Int J Cancer* **48**: 704–708

Chen S and Lee I (1984) Prognostic significance of morphology of tumor and retroperitoneal lymph nodes in epithelial carcinoma of the ovary. II. Correlation with survival. *Gynecol Oncol* **18**: 94–99

Desbaillets I, Tada M, De Tribolet N, Diserens AC, Hamou MF and Van Meir EG (1994) Human astrocytomas and glioblastomas express monocyte chemoattractant protein-1 (MCP-1) in vivo and vitro. *Int J Cancer* **58**: 240–247

Fidler I and Schroit A (1984) Synergism between lymphokines and muramyl dipeptide encapsulated in liposomes: in situ activation of macrophages and therapy of spontaneous cancer metastases. *J Immunol* **133**: 515–518

Graves DT, Barnhill R, Galanpoulos T and Antonoades HN (1992) Expression of monocyte chemoattractant protein-1 in human melanoma in vivo. *Am J Pathol* **140**: 9–14

Grimm MC, Elsbury SK, Pavli P and Doe WF (1996) Enhanced expression and production of monocyte chemoattractant protein-1 in iflammatory bowel disease. *J Leukoc Biol* **59**: 804–812

Haskill S, Becker S, Fowler W and Walton L (1982) Mononuclear-cell infiltration in ovarian cancer. I. Inflammatory-cell infiltrates from tumor and ascites material. *Br J Cancer* **45**: 728–736

Kuratsu J, Yoshizato K, Yoshimura T, Leonard EJ, Takeshima H and Ushio Y (1993) Quantitative study of monocyte chemoattractant protein-1 (MCP-1) in cerebrospinal fluid and cyst fluid from patients with malignant glioma. *J Natl Cancer Inst* **85**: 1836–1839

Leonard EJ and Yoshimura T (1990) Human monocyte chemoattractant protein-1 (MCP-1). *Immunol Today* **11**: 97–101

Mantovani A (1994) Tumor-associated macrophages in neoplastic progression: a paradigm for the in vivo function of chemokines. *Lab Invest* **71**: 5–16

Mazzuccheli L, Loetscher P, Kapeller A, Uguccioni M, Baggiolini M, Laissue JA and Mueller C (1996) Monocyte chemoattractant protein-1 gene expression in prostatic hyperplasia and prostate adenocarcinoma. *Am J Pathol* **149**: 501–509

Melani C, Pupa S, Stoppacciaro A, Menard S, Colnaghi M, Parmiani G and Colombo M (1995) An in vivo model to compare human leukocyte infiltration in carcinoma xenografts producing different chemokines. *Int J Cancer* **62**: 572–578

© 1999 Cancer Research Campaign

Negus R, Stamp G, Hadley YJ and Balkwill F (1997) Quantitative assessment of the leukocyte infiltrate in ovarian cancer and its relationship to the expression of C-C chemokines. *Am J Pathol* **150**: 1723–1734

Negus R, Stamp G, Relf M, Burke F, Malik S, Bernasconi S, Allavena P, Sozzani S, Mantovani A and Balkwill F (1995) The detection and localization of monocyte chemoattractant protein-1 (MCP-1) in human ovarian cancer. *J Clin Invest* **95**: 2391–2396

Negus R, Turner L, Burke F and Balkwill F (1998) Hypoxia down-regulates MCP-1 expression: implications for macrophage distribution in tumors. *J Leukoc Biol* **63**: 758–765

Peng L, Shu S and Krauss JC (1997) Monocyte chemoattractant protein-1 inhibits the generation of tumor-reactive T cells. *Cancer Res* **57**: 4849–4854

Schall T (1991) Biology of the RANTES/SIS cytokine family. *Cytokine* **3**: 165–183

Valkovic T, Lucin K, Krstulja M, Dobi-Babic R and Jonjic N (1998) Expression of monocyte chemoattractant protein-1 in human invasive ductal breast cancer. *Pathol Res Pract* **194**: 335–340

*© 1999 Cancer Research Campaign*
